# *Candida*-Induced Infective Endocarditis With Large Vegetation on a Bicuspid Aortic Valve in an Immunocompetent Patient

**DOI:** 10.1155/cric/1447191

**Published:** 2025-09-25

**Authors:** Mohamed Omar Hassan, Can Baba Arın, Said Abdirahman Ahmed, Ishak Ahmed Abdi, Ahmed Shafie Aden, Mohamed Osman Omar Jeele, Ahmed Elmi Abdi

**Affiliations:** ^1^Cardiology Department, Mogadishu Somali Türkiye Training and Research Hospital, Mogadishu, Somalia; ^2^Cardiology Department, Health Science University, Dr. Siyami Ersek Thoracic and Cardiovascular Surgery Training and Research Hospital, Istanbul, Turkey; ^3^Internal Medicine Department, Mogadishu Somali Türkiye Training and Research Hospital, Mogadishu, Somalia

**Keywords:** antifungal treatment, bicuspid aortic valve, fungal endocarditis, immunocompetent

## Abstract

**Introduction:** Infective endocarditis (IE) is a life-threatening condition caused by microbial infection of the heart valves or endocardium. Fungal IE, predominantly caused by the *Candida* species, accounts for less than 2% of IE cases, and is usually associated with immunosuppression or other risk factors. This case report describes an unusual instance of *Candida albicans* endocarditis with extensive aortic valve vegetation in an immunocompetent patient, highlighting the challenges in diagnosis and management.

**Case Report:** A 26-year-old active-duty soldier with no history of chronic illness presented with dyspnea, peripheral edema, and oliguria. Examination revealed a diastolic murmur, anemia, acute renal failure, and elevated inflammatory markers. Imaging showed cardiomegaly, pleural effusion, and a bicuspid aortic valve with large, mobile vegetations. Blood cultures confirmed *C. albicans*, and antifungal therapy with fluconazole was initiated. Despite aggressive medical management, including hemodialysis, the patient's condition deteriorated, and due to hemodynamic instability, surgery was not feasible. The patient unfortunately succumbed to complications.

**Discussion:** *Candida* endocarditis is uncommon in immunocompetent individuals; biofilms enhance resistance against antifungal therapy and the immune response, even in immunocompetent individuals. The absence of conventional risk factors poses diagnostic challenges. The case also underscores the rapid progression and poor prognosis of fungal IE, particularly with extensive vegetations and hemodynamic instability.

**Conclusion:** *Candida* endocarditis is a rare but severe condition, even in healthy individuals. This case emphasizes the importance of early recognition, comprehensive management, and further research to optimize outcomes in fungal IE.

## 1. Introduction

Infective endocarditis (IE) is a critical and sometimes fatal disease marked by the infection of the heart valves or endocardial surface, usually induced by bacteria, fungus, or other pathogens. Aortic valve IE is particularly significant among the various forms of endocarditis due to its potential for severe complications, such as vegetations, abscesses, and pseudoaneurysms, which can result in considerable morbidity and mortality if not promptly diagnosed and treated [1] [2]. The pathogenesis of aortic valve endocarditis frequently involves the colonization of the valve by microorganisms, resulting in the loss of valvular structures and adjacent tissues, which can lead to problems such as heart failure, embolic events, and systemic infections [3]. The clinical presentation of IE can vary significantly, from nonspecific symptoms like fever and malaise to more severe presentations, including sudden heart failure and embolic events impacting several organs [4]. In immunocompetent individuals, the incidence of IE may be lower than in those with predisposing factors such as prosthetic valves, congenital heart defects, or intravenous drug use; nonetheless, it is an essential diagnosis to consider in patients exhibiting relevant symptoms [5]. Identification of the causal organism determines antibiotic treatment and surgical intervention, especially in instances with extensive vegetations or abscesses [6]. A very uncommon kind of IE, especially in immunocompetent people, is *Candida* infectious endocarditis (CIE). Less than 2% of all instances of IE are thought to be fungal endocarditis, including those brought on by *Candida* species [7]. The unusual *Candida*-induced endocarditis with extensive aortic valve vegetation in an immunocompetent patient makes this case special.

## 2. Case Report

A 26-year-old male, an active-duty soldier with no history of chronic diseases, past medical admissions, or surgeries, presented to the emergency department with complaints of progressive shortness of breath, lower limb swelling, and reduced urine output. During the physical examination, significant bilateral pitting edema was observed in the pretibial area. A pronounced diastolic murmur was detected in the aortic region, accompanied by a systolic murmur radiating to the axilla. The lung examination indicated dull percussion bilaterally and diminished breath sounds in the basal areas.

Laboratory studies indicated anemia with a hemoglobin concentration of 10.8 g/dL. Renal function tests revealed markedly increased creatinine (3.7 mg/dL) and urea (178 mg/dL), suggesting acute kidney damage. A urine sample revealed significant leukocytosis, and severe metabolic acidosis was detected. Inflammatory markers were significantly high, with a C-reactive protein (CRP) level of 56 mg/L and procalcitonin level of 11.6 ng/mL.

**Figure 1:**
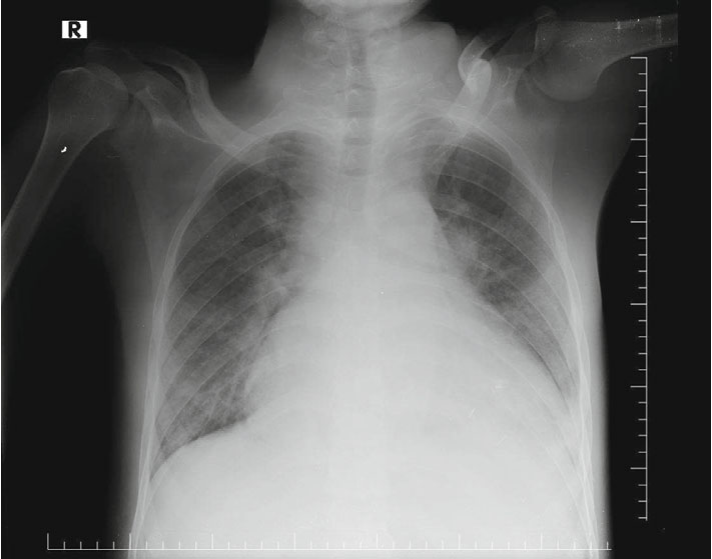
A chest X-ray demonstrated bilateral pleural effusion and cardiomegaly.

**Figure 2:**
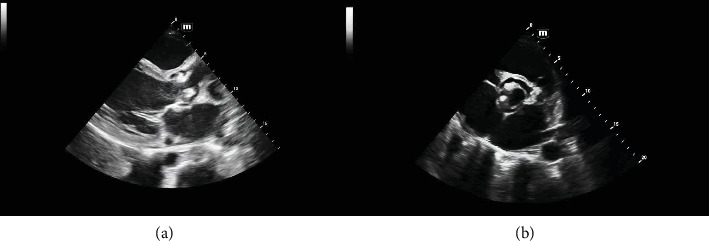
Transthoracic echocardiography revealing. (a) Large vegetations attached to both aortic valve leaflets in the long axis view. (b) Bicuspid aortic valve with the vegetation in the short axis view.

**Figure 3:**
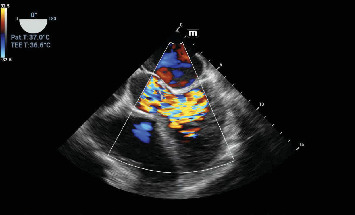
Transesophageal echocardiography confirmed a significant aortic regurgitation, with no indications of aortic root abscess or aneurysmal rupture.

A chest X-ray demonstrated bilateral pleural effusion and cardiomegaly (Figure 1). Transthoracic echocardiography revealed substantial vegetation affecting the bicuspid aortic valve leaflets (Figure 2). Left ventricular function was preserved (EF: 56%). The LV size was mildly dilated. Vegetations measured approximately 12 × 14 mm. Subsequent assessment using transesophageal echocardiography verified significant aortic regurgitation, with no indications of aortic root abscess or aneurysmal rupture (Figure 3).

The patient was brought to the inpatient department, and three blood culture samples and one urine culture sample were collected to determine the underlying illness. Urgent hemodialysis was commenced due to renal failure, congestion, and metabolic acidosis. The patient was started on fluconazole as an antifungal medication and received the full dose without any response. Despite rigorous medical intervention, the patient continued to exhibit hemodynamic instability. Blood and urine cultures demonstrated the presence of *Candida albicans*, an atypical result. Subsequent tests were performed to exclude immunocompromised states or autoimmune illnesses, all of which had negative results.

Surgical intervention was considered difficult due to the patient's critical state. Regrettably, the patient passed away from his illness shortly thereafter. This case underscores the fast advancement and unfavorable outlook linked to fungal IE compounded by significant aortic regurgitation, renal failure, and hemodynamic instability.

## 3. Discussion

This case report of *Candida*-induced urosepsis and IE resulting in significant aortic valve vegetation in an immunocompetent patient exhibits various unique characteristics that differentiate it from previous similar researches. The majority of *Candida* endocarditis occurrences occur in individuals with preexisting immunosuppression, including those receiving chemotherapy, infected with HIV, or possessing other notable comorbidities [8]. In contrast, the patient in this case report had no immunocompromising diseases, raising concerns about how such a serious illness could arise in a healthy person. The special pathogenicity of *Candida* species helps to explain the visible massive aortic valve vegetation in this case. These organisms are well known for creating biofilms on heart valves and other surfaces, which can cause notable vegetative development [9].

Biofilm production not only enhances the organism's adhesion to the valve but also shields it from the host's immune response and antifungal therapies, hence accelerating disease progression [10]. The presence of extensive vegetation can lead to considerable hemodynamic instability due to valvular insufficiency, as seen in this example when the patient had severe aortic valve regurgitation [11]. Furthermore, a bicuspid aortic valve markedly increases the chance of developing IE, particularly fungal endocarditis, as it is classified as a high-risk valve due to its atypical structure, rendering it more vulnerable to infection than a normal tricuspid aortic valve [12]. Fungal endocarditis therapy is difficult to determine due to a lack of clinical trial evidence. Health, hemodynamic stability, valvular damage, and life expectancy should determine treatment. Antifungal medication and surgery are often needed [13].

This case illustrates a rare instance of *Candida* endocarditis in an immunocompetent patient without identifiable fungal risk factors [14]. It also illustrates an unusual position for an aortic valve vegetation, observed on both leaflets of the aortic valve. Regrettably, our patient experienced an unfavorable outcome.

## Data Availability

Data supporting the findings of this case report are not publicly available due to concerns regarding patient privacy and confidentiality. Further details may be available from the corresponding author upon reasonable request and with appropriate ethical approvals.
